# Associations of CTLA4 Gene Polymorphisms with Graves' Ophthalmopathy: A Meta-Analysis

**DOI:** 10.1155/2014/537969

**Published:** 2014-07-09

**Authors:** Pengfei Du, Xiaojie Ma, Changjiang Wang

**Affiliations:** ^1^Department of Endocrinology, The Second Affiliated Hospital of Jiaxing University, Jiaxing 314000, China; ^2^Department of Clinical Pharmacy, The Second Affiliated Hospital of Jiaxing University, Jiaxing 314000, China

## Abstract

Many studies have established that T-lymphocyte antigen-4 (CTLA4) is a susceptible gene for Graves' disease (GD). Also many studies showed the association between the CTLA4 exon-1 49A/G polymorphism and the risk of developing Graves' ophthalmopathy (GO) in GD patients. But those results were inconsistent. In recent years many new studies were published which helped to shed light on the relationship of CTLA4 SNP49 with GO. So we performed the meta-analysis to explore the association between the SNP49 and GO susceptibility in GD patients. Studies up to February 29, 2012, were searched by using PubMed. The odds ratio was used to evaluate the strength of the association. Altogether 12 case-control studies involving 2,505 participants were included in the meta-analysis. Results showed that the G allele was related to the increased risk of GO compared with the A allele under allelic genetic model (OR = 1.14, 95% CI: 1.14–1.72, *P* = 0.001) in European subgroup. No publication bias was detected. Our results showed that the SNP49 polymorphism of CTLA4 gene was related to increased risk of GO.

## 1. Introduction

Graves' disease is a thyroid autoimmune disorder with 25–50% individuals having ophthalmopathy which is called thyroid-associated ophthalmopathy (TAO) or GO. The clinical manifestations of GO can mostly be explained by the increased volume of extraocular muscles and orbital connective tissues [1]. The symptoms of GO progress in some patients which eventually will lead to blindness. Though many studies have established the role of T cells in GO development [2–4], the understanding of the mechanism of GO is still poor. More effective therapy may develop on a better understanding of the T cell-fibroblast interaction.

Human CTLA-4 gene, located on 2q33, encodes a molecule which plays an important role in the downregulation of CD28 interaction with the ligands on the surface of antigen-presenting cells (APCs). The important inhibitory role of CTLA-4 in T-cell function has made it be one candidate gene when exploring autoimmune diseases.

Many reports have established the association of CTLA-4 with GD [5–9]. The CTLA-4 exon-1 +49A/G polymorphism has been found to be associated with the development of GO in GD patients in some studies but with inconsistent results [10–24].

One meta-analysis exploring the association of CTLA-4 gene polymorphism with GO was performed in 2006 which found no definite result [21]. Many new researches have been done since 2006 which will help to clarify the association. So we performed the meta-analysis with more case-control study results.

## 2. Methods

### 2.1. Search Strategy

We searched PubMed up to February 29, 2012, for all possible publications on the association between the CTLA-4 49A/G polymorphism and GO. Search terms were used as follows: “Cytotoxic T-lymphocyte associated antigen-4 or CTLA4,” “Graves' disease,” and “ophthalmopathy.” We also manually searched all reference lists of the relevant articles for additional papers. Language was limited to English. All eligible studies met the following criteria: (1) case-control design investigating the associations of CTLA4 49A/G polymorphism and GO; (2) providing genotype distribution information in both cases and controls or provided odds ratio (OR) with 95% confidence interval (95% CI) (or sufficient data that allowed us to calculate these). When a study reported results on different subpopulations, we treated each subpopulation as a separate comparison in the meta-analysis. For overlapping data, the most complete or recent study was included.

### 2.2. Data Extraction

Two authors (Pengfei Du and Changjiang Wang) independently extracted data and reached consensus on all items. The following information was sought from each report: authors, journal and year of publication, country of origin, selection and characteristics of GO cases and controls, demographics, ethnic group of the study population, eligible and genotyped cases and controls, and number of cases and controls for the genotype. For studies including subjects of different ethnic groups, data were extracted separately for each ethnicity whenever possible.

### 2.3. Statistical Analysis

The primary analysis compared GO cases with controls for the frequency of G versus A alleles. This analysis aims to detect overall differences. Pooled OR with 95% CI was used to evaluate the strength of the associations of CTLA-4 49A/G polymorphism and GO. When zero events occurred, we treated this problem by adding 0.5 to all the 2 × 2 cells of the contingency table to calculate the study-specific OR just as implemented in RevMan software. We examined the association of 49A/G polymorphism and GO for allelic contrast (G versus A). Furthermore, we conducted subgroup analyses by stratifying ethnicity into Europeans and Asians separately. A *Z* test was used to determine the significance of the pooled OR and *P* value <0.05 was considered significant. The between-study heterogeneity across all eligible comparisons was estimated using the chi-square based Q statistic [25]. Heterogeneity was considered significant for *P* < 0.10. Data were combined using both fixed-effects (Mantel-Haenszel) and random effects (DerSimonian and Laird) models [26]. Random effects incorporate an estimate of the between-study variance and tend to provide wider confidence intervals, when the results of the constituent studies differ among themselves. In the absence of between-study heterogeneity, the two methods provide identical results. Random effects are more appropriate when heterogeneity is present [26]. Inverted funnel plots and the Begg-Mazumdar publication bias diagnostic (nonparametric *τ* correlation coefficient) [27] evaluated whether the magnitude of the observed association was related to the variance of each study; that is, whether large studies gave different results compared with smaller ones. All the statistical analyses were performed using RevMan5.0 software (the Cochrane Collaboration, Oxford, England). All *P* values were two-sided.

## 3. Results

### 3.1. Data Summary

The primary literature search yielded 17 papers [10–24, 28, 29]. After further scrutiny, 12 potential relevant papers were identified. Of these papers, five papers were excluded for the following reasons: two papers include overlapping data [10, 29] with another two studies, and only the detailed one was selected [11, 21]; three studies [12, 14, 28] were not included because their genotype or allele distribution information was not available. One of the eligible studies [19] comprised subjects of two different racial descents, so it was evaluated as two studies. Finally, a total of 12 papers and 13 separate comparisons were included in our meta-analysis [11, 13, 15–24]. The general characteristics of the selected studies are shown in Table 1. Controls were GD patients without GO. These 12 papers all evaluated the association of 49A/G polymorphism with GO. Five studies were conducted in Asian populations [15, 19, 21–23] and the others were in European population. The sample size of individual studies ranged from 33 to 222. Polymerase chain reaction-restriction fragment-length polymorphism (PCR-RFLP), DCFH (dual-color fluorescence hybridization), and minisequencing were used as genotyping methods.  Table 2 shows the genotype frequencies in included studies.

### 3.2. Association of the 49A/G Polymorphism with GO

**Figure 1:**
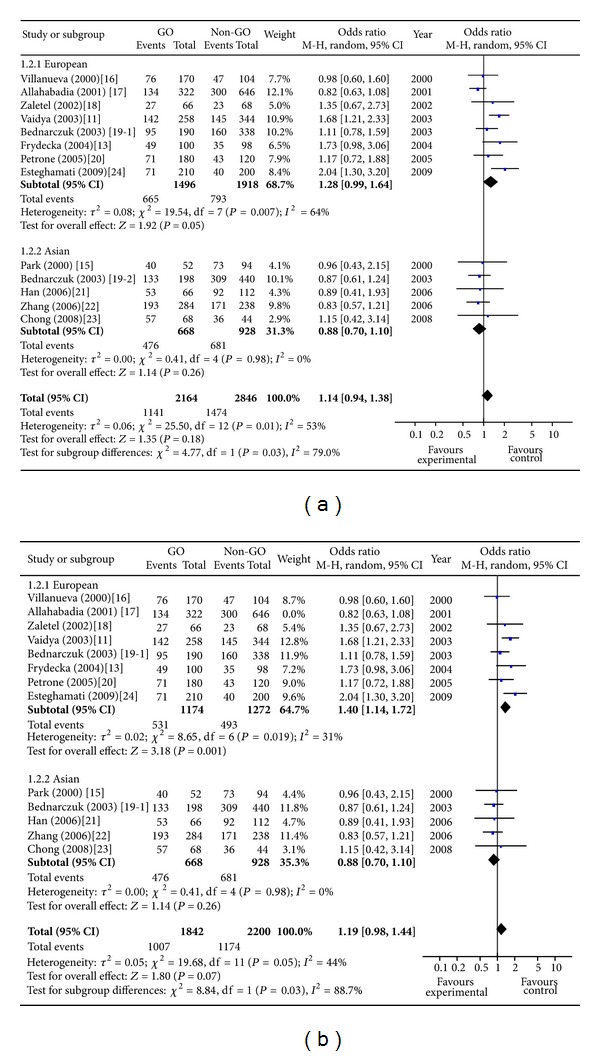
Meta-analysis for the effect of the G allele versus the A allele on the risk of GO in GD patients. Each comparison is presented by the name of the first author and the year of publication. The point estimate of the odds ratio and the accompanying 95% confidence interval (CI) are shown. “Total” represents the summary random effects estimation for the comparison along with the respective 95% confidence interval. Values above 1 denote an increased risk for GO with the G allele.

**Figure 2:**
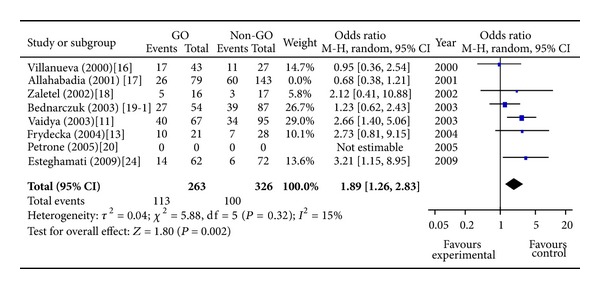
Meta-analysis for the effect of the GG versus the AA genotype on the risk of GO in GD patients in European subgroup. Each comparison is presented by the name of the first author and the year of publication. The point estimate of the odds ratio and the accompanying 95% confidence interval (CI) are shown. “Total” represents the summary random effects estimation for the comparison along with the respective 95% confidence interval. Values above 1 denote an increased risk for GO with the GG genotype.

**Figure 3:**
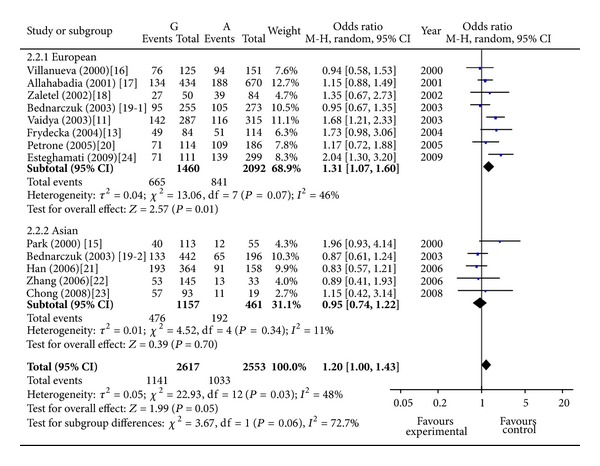
Meta-analysis for the effect of the CTLA4 49A/G allele in G carriers and in A carriers on the risk of GO in GD patients. Each comparison is presented by the name of the first author and the year of publication. The point estimate of the odds ratio and the accompanying 95% confidence interval (CI) are shown. “Total” represents the summary random effects estimation for the comparison along with the respective 95% confidence interval. Values above 1 denote an increased risk for GO with the G allele.

A total of 12 studies concerning the 49A/G polymorphism included 2,505 individuals (1,082 cases and 1423 controls). The pooled frequency of G allele was 68.1% and 51.8% for cases and controls, respectively. Two studies suggested an at-risk effect of G allele [11, 24] and the others did not produce significant results. The overall analysis for evaluating the association of 49A/G polymorphism with GO under allelic model showed that there was large heterogeneity between these 13 studies (*P* heterogeneity = 0.01), and the random effects pooled OR was not significant: OR = 1.14, 95% CI: 0.94–1.38, *P* = 0.18 (Figure 1). One study was excluded from the meta-analysis because of the contribution to the heterogeneity in the European subgroup (random OR = 1.40, 95% CI 1.14–1.72, *P* = 0.001, *P* heterogeneity = 0.19) [17]. Sensitivity analyses evaluating the influence of single studies on overall risk estimate by omitting one study in each turn did not materially alter the overall effect size, with a range from OR = 1.32 (95% CI 1.09–1.60) to OR = 1.48 (95% CI 1.19–1.85). The GG genotype is associated with increased risk of GO in European subgroup (random OR = 1.89, 95% CI 1.26–2.83, *P* = 0.002, *P* heterogeneity = 0.32) (Figure 2). The G carrier was more likely to have GO than A carrier in European subgroup (random OR = 1.31, 95% CI 1.07–1.60, *P* = 0.01, *P* heterogeneity = 0.01) (Figure 3).

## 4. Discussion

The current meta-analysis including 12 case-control studies was in an effort to clarify the relationship between CTLA-4 gene polymorphism and GO susceptibility. The overall results indicated that the CTLA-4 49A/G polymorphism was associated with susceptibility of GO which was more significant in European population. Although obvious heterogeneity was detected for 49A/G associations, sensitivity analyses did not materially alter the overall and subgroup results under different genetic models, indicating that the results were stable and reliable.

The polymorphism of CTLA-4 had been suggested in many diseases which included Addison's disease, autoimmune hypothyroidism, and rheumatoid arthritis. The CTLA-4 49A/G SNP in exon-1 leads to the substitution of Ala with Thr in the signal peptide part which was reported to cause misprocessing of CTLA-4 in the ER resulting in less efficient glycosylation and diminished surface expression of CTLA-4 protein [30]. Studies have demonstrated that the longer repeats of the 3′UTR microsatellite are associated with reduced CTLA-4 inhibitory function [31]. Some workers have shown association between the G allele of 49A/G and reduced control of T-cell proliferation [9, 32]. One study found an association between the protective allele A of the 49A/G SNP, which is associated with increased CTLA-4 function/expression, and interferon induced thyroiditis [33]. Hence, we postulated that, under most cases of spontaneous autoimmunity, alleles associated with reduced CTLA-4 function and/or expression could lead to increased activation of T cells thereby triggering autoimmunity.

The association of CTLA-4 49A/G SNP with GO was more significant in European population but not in Asian population. The CTLA4 49A/G SNP shows the same effect on the GO development with different significance. The results are confusing but not conflicting. This can be explained by the following reasons. Firstly, both GD and GO are multigenic conditions. It is unlikely to have one major susceptibility gene contributing to the development of GO. In the studies of Xu et al., they transiently transfected a T-cell line with a CTLA-4 construct harboring either the G or the A allele of the 49A/G SNP and found no difference in CTLA-4 expression and/or function harboring the A or the G allele [34]. It is likely that the 49A/G SNP did not directly affect the T-cell activation but may in LD with some major effect alleles. Secondly, the impact of smoking on GO was not specifically considered in the association studies. As was reported, the odds ratio for both current smokers and exsmokers was high and the GO was more severe in smokers than in nonsmokers [35–37].

In conclusion, our analysis first intensified the association between 49A/G and GO susceptibility, which showed that the G allele is a risk factor for GO. More risk factors should be considered in future association studies of the CTLA-4 SNP with GO. The gene-gene and gene-environment interaction should also be taken into consideration in future research. The identification of the candidate gene and understanding of the mechanisms by which they cause disease will identify those individuals at risk for developing GO in the future. Treatment will evolve towards more preventive strategies. The treatment will be personalized by targeting the specific pathways that are most contributory to GO development. We believe that the future will witness the engagement of novel molecules, such as altered peptide ligands [38] and specific monoclonal antibodies, to modulate specific autoimmune pathways in affected individuals, thus treating the cause of the disease.

## Figures and Tables

**Table 1:** General characteristics of the selected studies in the meta-analysis.

Ethnic	Country	SNP genotyping	Grouping method		Eligible subjects		First author (year) [reference]
			GO	Non-GO	GO	Non-GO	
European	USA	RFLP	NOSPEC3–6	No clinical feature	85	52	Villanueva (2000) [16]
	UK	RFLP	NOSPEC3–6	NOSPEC < 3	161	323	Allahabadia (2001) [17]
	Slovenia	RFLP	Clinical features	No clinical feature	33	34	Zaletel (2002) [18]
	Iran	RFLP	NOSPEC3–6	NOSPEC < 3	105	100	Esteghamati (2009) [24]
	Poland	RFLP	NOSPEC3–6	No clinical feature	95	169	Bednarczuk (2003) [19]
	UK	RFLP	NOSPEC3–6	NOSPEC < 3	124	168	Vaidya (2003) [11]
	Poland	Minisequencing	NOSPEC3–6	NOSPEC < 3	50	49	Frydecka (2004) [13]
	Italy	RFLP	NOSPEC2–6	NOSPEC < 3	90	60	Petrone (2005) [20]

Asian	China	DCFH	NOSPEC3–6	NOSPEC < 3	142	119	Han (2006) [21]
	China	RFLP	NOSPEC3–6	No clinical feature	33	56	Zhang (2006) [22]
	China	RFLP	NOSPEC2	NOSPEC3, 4	34	22	Chong (2008) [23]
	Japan	RFLP	NOSPEC3–6	No clinical feature	99	220	Bednarczuk (2003) [19]
	Korea	RFLP	Clinical feature	No clinical feature	26	47	Park (2000) [15]

**Table 2:** Distribution of CTLA4 49A/G alleles among GO and controls in the included studies.

First author	Country	A/A (*n*)		A/G (*n*)		G/G (*n*)	
		GO	Non-GO	GO	Non-GO	GO	Non-GO
Villanueva (2000) [16]	USA	26	16	42	25	17	11
Allahabadia (2001) [17]	UK	53	83	82	180	26	60
Zaletel (2002) [18]	Slovenia	11	14	17	17	5	3
Esteghamati (2009) [24]	Iran	48	66	43	28	14	6
Bednarczuk (2003) [19]	Poland	27	48	41	82	27	39
Vaidya (2003) [11]	UK	27	61	62	77	40	34
Frydecka (2004) [13]	Poland	11	21	29	21	10	7
Petrone (2005) [20]	Italy	NA	NA	NA	NA	NA	NA
Han (2006) [21]	China	18	14	55	39	69	66
Zhang (2006) [22]	China	1	1	11	18	21	37
Chong (2008) [23]	China	1	0	9	8	24	14
Bednarczuk (2003) [19]	Japan	12	16	41	99	46	105
Park (2000) [15]	Korea	1	3	10	15	15	29

NA: not available.
